# Exploring cross-sectional associations between common childhood illness, housing and social conditions in remote Australian Aboriginal communities

**DOI:** 10.1186/1471-2458-10-147

**Published:** 2010-03-20

**Authors:** Ross Bailie, Matthew Stevens, Elizabeth McDonald, David Brewster, Steve Guthridge

**Affiliations:** 1 Menzies School of Health Research, Institute of Advanced Studies, Charles Darwin University, Darwin, Northern Territory, Australia; 2 School of Medicine, James Cook University, Cairns, Queensland, Australia; 3 Health Gains Planning Division, Nth Territory Government Department of Health and Families, Darwin, Nth Territory, Australia

## Abstract

**Background:**

There is limited epidemiological research that provides insight into the complex web of causative and moderating factors that links housing conditions to a variety of poor health outcomes. This study explores the relationship between housing conditions (with a primary focus on the functional state of infrastructure) and common childhood illness in remote Australian Aboriginal communities for the purpose of informing development of housing interventions to improve child health.

**Methods:**

Hierarchical multi-level analysis of association between carer report of common childhood illnesses and functional and hygienic state of housing infrastructure, socio-economic, psychosocial and health related behaviours using baseline survey data from a housing intervention study.

**Results:**

Multivariate analysis showed a strong independent association between report of respiratory infection and overall functional condition of the house (Odds Ratio (OR) 3.00; 95%CI 1.36-6.63), but no significant association between report of other illnesses and the overall functional condition or the functional condition of infrastructure required for specific healthy living practices. Associations between report of child illness and secondary explanatory variables which showed an OR of 2 or more included: for skin infection - evidence of poor temperature control in the house (OR 3.25; 95%CI 1.06-9.94), evidence of pests and vermin in the house (OR 2.88; 95%CI 1.25-6.60); for respiratory infection - breastfeeding in infancy (OR 0.27; 95%CI 0.14-0.49); for diarrhoea/vomiting - hygienic state of food preparation and storage areas (OR 2.10; 95%CI 1.10-4.00); for ear infection - child care attendance (OR 2.25; 95%CI 1.26-3.99).

**Conclusion:**

These findings add to other evidence that building programs need to be supported by a range of other social and behavioural interventions for potential health gains to be more fully realised.

## Background

Children in remote Australian Aboriginal communities experience exceptionally high rates of common childhood infections including otitis media, skin and respiratory infections and gastroenteritis [1-4]. These infections have serious consequences, including high rates of chronic suppurative otitis media [3], bronchiectasis [5], rheumatic heart disease [6,7] and impaired growth and development [2,8] permanent hearing loss [9] and consequent poor educational outcomes [10]. These infections in childhood contribute to high rates and early onset of chronic disease in adulthood [11] and to the 17 year gap in life expectancy between Indigenous (Aboriginal and Torres Strait Islander peoples) and other Australians [10].

Poor housing conditions are widely regarded as being an important underlying factor for these poor Indigenous child health outcomes [5] Housing impacts on health through two main mechanisms; i) poor housing conditions; and ii) overcrowding due to a shortage of housing. Poor housing conditions facilitate the transmission of infection among children through: unhygienic, poorly functioning or inadequate water and sanitation technology and systems; poor ventilation; damp; mould; and extremes of temperature [12-14]. Poor and old housing infrastructure provides breeding sites for disease causing vermin such as cockroaches and rats. Housing that does not enable residents to safely store and prepare food places children at greater risk of diarrhoeal diseases. Overcrowding leads to increased interpersonal contact between residents. This promotes the spread of infections, especially respiratory disease and scabies. This increased interpersonal contact may aggravate stress associated with poor housing conditions and other day-to-day stressors (lack of privacy, loss of control, high demand, noise, lack of sleep) [15-18], and has been associated with raised levels of stress and poor mental health (physical and psychological withdrawal, aggression, depression) [19-22]. In many remote Aboriginal communities in the Northern Territory the effects of overcrowding and poor household infrastructure combine, adding to the significance of the risks posed by housing conditions to health, with children and the elderly being the most vulnerable to these risks [12].

There is limited previous epidemiological research that has aimed to gain insight into the complex web of causative and moderating factors that links housing conditions to a variety of poor health outcomes [14,23]. In resource-poor contexts research focuses more on the introduction of water and sanitation technology and hygiene promotion to prevent serious diarrhoeal and respiratory infections [24]. Among disadvantaged communities in resource-rich settings, the focus is less on preventing common childhood infections but rather on preventing and treating conditions such as asthma and the effects of social and/or emotional stress [25]. Few studies of housing and health have measured and adjusted for the range of relevant confounders [12], or have adequately addressed the range of factors that may need to be considered in a multifaceted intervention on housing for health improvement [14]. Few intervention or follow-up housing studies have been completed [12].

The construction of additional housing in remote communities has been a priority strategy of Government to improve Aboriginal child health for some years [26]. Efforts in this area have received a substantial funding boost through recent inter-governmental agreements [27]. While there is a lack of research to inform the current policy and program initiatives to improve housing and health in the Australian Indigenous setting, our systematic review of hygiene and public health interventions to improve child health in these communities highlights the requirement for multifaceted interventions.

A complex mix of political, economic, social and physical factors underlies the poor living conditions and subsequent poor health of children in remote communities [28]. This mix of factors presents a challenge for research which aims to discern the significance of the various factors, and for policy and practice in developing and implementing multifaceted interventions that address the critical intervention points [28-30]. Housing programs continue to be developed in the absence of good information about the critical points for intervention by which housing programs can improve health.

This paper reports on baseline data from a study which aims to assess the impact of building programs on the occurrence of common childhood illness in remote Australian Aboriginal communities. The purpose of the paper is to provide insight into the social and environmental correlates, with a primary interest in the functional state of household infrastructure and carers' reports of a number of common childhood illnesses. The reason for the emphasis in this study on the functional state of infrastructure is that this has been the strongly predominant focus of housing programs[26,27]. The approach of assessing multiple exposures against multiple outcomes follows the principles of the Multiple Exposure Multiple Effects (MEME) Model [31], and aims to provide information that is more relevant to broader policy and program planning than can be achieved through studies focussed on specific exposures and specific outcomes.

## Methods

### Study setting

There are several hundred discrete Aboriginal communities in the Northern Territory (NT) that range in size from a single family group to 2500 people. Indigenous people make up almost 30% of the approximately 200,000 people living in the NT, and over 70% of these people (i.e. about 47,000 people) live in locations which are isolated by distance and terrain from the type of modern-day economic activity and services to be found in rural and regional Australian towns [32]. These remote Aboriginal communities developed largely in association with missionary, mining or agricultural activity during the colonial era. The communities are distant from significant sources of industrial pollution, and the major sources of pollution of the community environment are dysfunctional sanitation systems (or unhygienic child toileting practices [33]) and domestic human waste (litter, car bodies, discarded construction materials, burning of household rubbish). The people suffer significant disadvantage in health and socio-economic terms compared to the general Australian population [28]. The housing in these communities ranges from modern design and construction to formal but relatively crude brick and mortar and tin constructions to makeshift shelters. Houses are generally publically owned and are commonly poorly maintained with dysfunctional hardware (such as taps, sinks, and doors) [34]. The study focussed on ten communities with higher levels of planned construction in relation to community population and which reflected geographic spread of communities and architectural diversity.

### Study design

**Figure 1:**
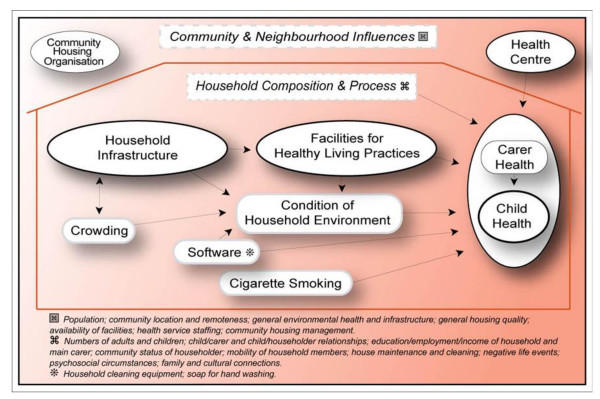
**Conceptual framework for housing and child health**.

The Housing Infrastructure and Child Health (HICH) study is built around the conceptual framework depicted in Figure 1 and on the implementation of housing construction programs in NT communities over the period 2003 and 2004. The primary focus of this study - the relationship between the functional state of infrastructure and child health status - is highlighted by the bold outlined ellipses across the middle of the framework. The wide range of other related influences on child health (potential confounders of the relationship between functional state of infrastructure and child health) and the relationships between these influences are reflected in the other broad constructs and the arrows shown in the framework. The processes for community engagement, development of survey forms, obtaining informed consent, conduct of the fieldwork and community feedback are described in detail in a previous publication [35]. In brief, all the houses in the ten communities where there was at least one child aged seven years or less were identified. Data collection included: structured interviewer administered surveys of the main carer for each child in this age group and of the main householder; a systematic detailed survey of the functional state of the household infrastructure; a survey of the general community environment; and an interview with a senior member of the community council or housing office.

### Outcome measures

These were obtained for five childhood illnesses (i. respiratory infection; ii. diarrhoea and/or vomiting; iii. ear infection; iv. scabies with or without skin infection (includes boils); and vi. skin infection (includes boils) with no scabies) that are common in remote communities by asking the primary carer if the child had an illness in the last 2 weeks. Specifically, the primary carer of the child was asked "*has *[the child's name] *had a *[illness]*in the last 2 weeks?*" For skin infection, the question was asked separately for scabies, skin sores and boils with two outcome measures coded post-survey. Questions about these outcomes were asked using the colloquial terms used by local community residents to describe these illnesses. Appropriate interpretation of these and all other survey questions was supported by employment of local community residents to assist the survey teams, and by piloting, standardisation and training of surveyors [35].

### Primary explanatory variables (Table 1)

**Table 1:** Primary explanatory variables: Functional state of infrastructure required for healthy living practices (HLPs)

Specific FHLP scores	Criteria for assessment of functional standard (pass/fail)
Wash children	Conditions required to pass depend on age of child:
	(1) For child <1 year: (i) bathroom basin, hot tap, cold tap, bench, door, electrical and general structure all functioning, or (ii) kitchen sink, hot tap and cold tap all functioning.
	(2) For child aged 1 to <3 years: (i) laundry trough, hot tap, cold tap, shelf, electricity, floor drainage and general structure all functioning, or (ii) the bathroom shower head, hot tap, cold tap, drainage, bench, electrical and general structure all functioning.
	(3) For child aged 3 to less than 7 years: bathroom shower head, hot tap, cold tap, drainage, bench, door, electrical and general structure all functioning.
	At least one item not functioning from any condition - fail

Wash clothes & bedding	Laundry trough, hot tap, cold tap, shelf, electricity, floor drainage and general structure all functioning - pass.
	At least one item not functioning - fail

Prepare & store food	Sink taps, sink, cold water flow, pantry, oven, stove top, cooking/eating utensils, bench, lights and electrical fittings, and kitchen general structure all functioning - pass.
	At least one item not functioning - fail

Remove human waste (toilet and drainage)	Conditions required to pass depend on age of child:
	(1) If child <1 year: toilet pan, cistern, water supply, drainage, bathroom basin and hot and cold taps all functioning.
	(2) If child aged 1 to <3 years: child toilet equipment available (e.g. potty - small plastic toilet) and toilet pan, cistern, water supply, drainage, electricity, general structure, bathroom basin and hot and cold taps all functioning.
	(3) If child aged 3 to less than 7 years: toilet door, electricity, general structure, toilet pan, cistern, water supply, drainage, bathroom basin, hot tap and cold tap all functioning.
	At least one item not functioning from any condition - fail

Remove waste water	Laundry, toilet and shower drainage all functioning - pass.
	At least one item not functioning - fail

Remove rubbish	Indoor and/or outdoor rubbish bins present - pass.
	Indoor and/or outdoor rubbish bins absent - fail

Boundary fence	Boundary fence present and intact (to control dust) - pass.
	Boundary fence absent or broken down - fail

Electricals	Supply in kitchen, laundry, toilet, main bedroom, 2^nd ^bedroom, switch box & earth all in safe condition & functioning - pass.
	At least one item not functioning - fail
**Overall FHLP score**	

Number of HLPs failed	0-2 failed
	3-8 failed

Composite variables reflecting the functional state of items of housing infrastructure that enabled residents to carry out healthy living practices (HLPs) were based on the approach used in previous housing development and research work [36-40]. The Failed Healthy Living Practices (FHLPs) score was based on an assessment of all items of infrastructure required for conducting each HLP (Table 1) along the lines specified in the national Indigenous Housing Guide [40]. For any HLP for which an item of infrastructure was not functioning at an adequate level (fully functional or requiring minor maintenance only), the infrastructure required for that HLP was scored as failed. The overall assessment of house function using this method is then based on the number of failed 'HLPs' and was completed during data analysis. The overall FHLP score reflects the number of HLPs for which the score was 'fail' (potential range 0-8).

### Secondary explanatory variables (Additional files 1, 2, 3 and 4)

These variables reflect measurable indicators of constructs in our conceptual framework (Figure 1). The 'socio-demographic' and 'socio-economic' variables (Additional files 1 and 2) are indicators of 'household composition and process'. The 'psychosocial' variables (Additional file 3) are indicators of carer social and emotional wellbeing as a potentially important influence on child health. These variables overlap to some extent with the 'household composition and process' construct. The 'health related behaviour and hygienic state of environment' variables (Additional file 4) include indicators of availability of household cleaning equipment and soap for personal hygiene; of exposure of young children to cigarette smoke; the protective effect of breastfeeding and of the hygienic condition of the household environment. Hygienic condition of the house was assessed on a 1 (best) to 7 (worst) Likert scale, with the 'expected average' as defined by experienced surveyors to achieve a score of 4. The repeatability of the measures on this score is high, with the great majority of measurements being within one point of the original score on the seven point Likert scale (paper in press).

Crowding is included among the 'socio-demographic variables' (Additional file 1) and is represented separately in the conceptual framework (Figure 1) because of the potential important direct effect on the general quality and functional state of household infrastructure and on the state of hygiene of the living environment. Community and neighbourhood level indicators (including those related to community housing organisations and community health centres) are not presented here because our preliminary analysis did not show significant associations with the child health outcomes that are the subject of this paper.

Two separate measures were used to assess carer mental health and wellbeing. The Negative Life Events Scale (NLES) was developed by the Australian Bureau of Statistics (ABS) in consultation with peak Aboriginal health bodies [41] to measure social and emotional well-being by measuring exposure to stressful life events [42], with the aim of providing a valid measure for Indigenous and non-Indigenous Australians. It has been used in recent national social and health surveys [43,44]. In a validation study using data from this study [45], we showed the measure to have good reliability and internal and external validity. The NLES measures a range of stressful life events (poor health, loss of a job, death of a family member, experience of actual or threatened violence, and trouble with the police). Through factor analysis we identified three factors: NLES-1: Seeing fighting, drug problems, alcohol problems, witness violence, and vandalism (alpha = 0.79); NLES-2: Gambling problems, serious accident, police trouble, someone close sent to jail, racism (alpha = 0.65); and NLES-3: Serious illness or disability, death of family member, and overcrowding (alpha = 0.53).

The Brief Screen for Depression (BSD) [46] produces a score out of 50. Respondents scoring greater than or equal to 25 were classified as depressed (alpha = 0.45), consistent with the approach used by the designers of this tool.

### Statistical analysis

Carer report data on each child health outcome provides a dichotomous measure which is suited to logistic regression modelling. The analysis was carried out in a hierarchical process due to the large number of explanatory variables and follows a broadly similar process to that used in the pilot study [38] and other similar studies [47]. Specifically, there were variables from socio-demographic (including crowding), socioeconomic and financial stress, psychosocial, health behaviour and hygiene domains, as well as the primary explanatory variables which consisted of composite measures of housing functionality (see Table 1). The following process was carried out for each of the five child health outcomes. First, bivariate associations were calculated between all explanatory variables and each health outcome, with those showing a moderate association (p < 0.20) retained for the next stage. The second stage was carried out separately for each domain (i.e. socio-demographic, psychosocial etc.) and involved including all variables retained from stage one into a multivariable model and applying backward elimination (removal set at p > 0.05). Due to the non-independence of the overall FHLP score with individual housing functionality measures only individual measures of housing functionality were included initially and backward elimination applied. The model was then run again using the overall FHLP score in place of specific FHLP scores. The third and final stage involved including all variables retained from stage two into a single multivariable model and again applying backward elimination to arrive at final models for each of the five outcomes. If the overall FHLP score was carried through then separate models were tested using the overall FHLP score in place of any specific FHLP scores. Plausible first order interactions were then tested and if significant, all possible contrasts were tested and significant contrasts are presented along with the final models. All confidence intervals were adjusted for clustering of children by community and dwelling using the Huber-White sandwich variance estimator [48]. Fisher Exact tests were also carried out to assess differences in explanatory variables for children excluded from the final multivariable models. All statistical analyses were carried out using Stata v9.2^©^.

Ethics approval for the study was obtained from the Top End and Central Australia Human Research Ethics Committees in-line with the requirements of the National Health and Medical Research Council of Australia.

## Results

The median reported population for the ten participating communities was 588 (range 250-1450), with 328 houses identified as being home to at least one child in the eligible age range. In 12 (4%) of these houses the householder declined any involvement, in 19 (6%) the householder was not available on at least three repeated visits and in 18 (5%) the householder agreed to be interviewed but refused the house survey. Interview and survey data were available from 279 (85%) houses with children in the eligible age range, and we obtained data on 618 individual children aged seven years or less who were living in these houses - i.e. 85% of the estimated total of 727 children in the eligible age range in these communities (based on surveys of 85% of houses of children in the eligible age range and assuming the same number of children on average in participating and non-participating households).

For the 618 children, carers reported each of the conditions of interest to have occurred within the two weeks preceding the survey as follows: skin infection (with no scabies) in 19.7%, scabies (with or without skin infection) in 17.1%; respiratory infection in 28.8%, diarrhoea and/or vomiting in 30.6%, and ear infection in 28.0% (percentages add up to more than 100% because some children had more than one condition reported). Complete data were available for all children for the outcome variables, while for the primary explanatory variables between 5% and 8% of children had missing data. Only a small proportion of children (<10%) had missing data for specific secondary explanatory variables (Additional file 1, 2, 3 and 4).

Unadjusted associations between primary explanatory variables and carer's report of each of the childhood illnesses are presented in Additional file 5. Statistically significant associations between carer's report of each of the child illnesses and poor infrastructure were found for: scabies and removal of rubbish and control of dust; diarrhoea and/or vomiting and preparation and storage of food; ear infection and toilet infrastructure and poor infrastructure overall. There were no other statistically significant associations between any of the measures of function of specific components of household infrastructure or of the overall state of household infrastructure and carer's report of skin infection or respiratory infection, although the general trend was towards poor infrastructure being associated with carer's reports of illness across all components of infrastructure and all illnesses. Children living in houses that were in poor overall condition (did not meet the requirements for effective conduct of three or more of the eight healthy living practices) tended to have more reported illness, with Odds Ratios (ORs) higher than 2 for four out of the five recorded illnesses, with one being statistically significant and two being of borderline statistical significance. A trichotomous variable was also created for the number of healthy living practices failed (0-2, 3-5, and 6-8) to investigate dose response or non-linear associations with the outcomes. Odds Ratios for children in houses failing 6 to 8 HLPs did not differ from those in the in houses scoring 3 to 5 compared with the reference group (scores 0 to2), so we proceeded to use the dichotomous variable in all analyses.

Unadjusted associations between the secondary explanatory variables and carer's report of each of the childhood illnesses are presented in Additional files 1, 2, 3 and 4. The variables for which there were associations with more than one of the reported illnesses were age (more reports of skin infection in 1-2 year age group and 3-7 year age group; more reports of ear infection in 1-2 year age group; fewer reports of diarrhoea and/or vomiting in 3-7 year age group); male sex (more reports of respiratory infection and diarrhoea and/or vomiting); grandparent relationship between householder and the child (more reports of skin infection, scabies, respiratory infection, and diarrhoea and/or vomiting); increased report of negative life events (factor 2) (respiratory infection and diarrhoea and/or vomiting); number of people who smoke inside the house (skin infection, scabies, and diarrhoea and/or vomiting); poor hygienic condition of the bedding and sleeping area (diarrhoea and/or vomiting, ear infection); and overall hygienic condition of the house (skin infection).

The strongest associations of reported illnesses and the secondary explanatory variables (ORs of 3 or more) are seen between ear infection and child age (highest reporting in 1-2 year age group); skin infections and large numbers of adults in the house; scabies and larger numbers of people smoking indoors; ear infection and poor hygienic condition of bedding and sleeping areas; and skin infections and intermediate scores for evidence of adequate temperature control.

The results of multivariable models for each of the reported illnesses are presented in Additional file 6. Children with missing data for the variables included in any of these models did not differ from the rest of the children in terms of age, gender, child mobility, presence of carer's spouse, relationship to householder or carer, carer's education, financial security, social support, psychosocial status, householder's community status, history of breastfeeding, or presence of soap or cleaning equipment. While children with missing data did differ on some variables for some models there was no clear pattern for these children in terms of advantage or disadvantage in relation to the primary or secondary explanatory variables. For the final multivariate models, between less than 1% and 13% of children were excluded because of missing data.

Explanatory variables which showed an independent significant association with carer's report of *skin infection *were a poor score for evidence of pests and vermin in the house, and an intermediate score for evidence that the house had adequate temperature control facilities; for *scabies*: a poor score for evidence of pests and vermin, and a protective effect of the carer living with her/his spouse; for *respiratory infection*: a poor overall score for the functional state of house infrastructure, younger age of child (<1 year vs. 3-7 years), carer positive screen for depression, and a protective effect for breastfeeding; for *diarrhoea and/or vomiting*: younger age of child (<1 year and age 1-2 years vs. 3-7 years), male sex, carer report of negative life events (factor 2), absence of soap in the house, and an intermediate score for food preparation and storage facilities; and for *ear infection*: age of child (age 1-2 years vs. <1 year), and day care attendance (Additional file 6).

The variables for which there was an association with more than one of the reported illnesses were age, and a poor score for evidence of pests and vermin in the house (skin infection and scabies). The strongest associations of reported illnesses and the explanatory variables (ORs of 3 or more; or of 0.3 or less for protective factors) are seen between respiratory infection and overall functional condition of the house and breastfeeding; diarrhoea and/or vomiting and ear infection and child age (highest reporting in 1-2 year age group); skin infections and intermediate scores for evidence of adequate temperature control (Additional file 6).

## Discussion

The reporting of common childhood illness was associated with indicators which relate to a number of the constructs presented in our conceptual framework for housing and child health. In the multivariate analysis, the functional state of infrastructure required for conducting healthy living practices was associated with increased reporting of respiratory infections, but not with reporting of the other childhood illnesses. This association was shown for the indicator of overall function of household infrastructure, and not for indicators of functional state of infrastructure relating to specific healthy living practices. This finding points to the importance of general improvement in the functional state of household infrastructure across the facilities required for a range of healthy living practices rather than a focus on specific aspects of infrastructure believed to be important in preventing respiratory infections [36]. While the association between the functional state of infrastructure and reporting of childhood illnesses was shown to be significant only in the multivariate model for respiratory infections, the relatively high ORs seen in the bivariate (unadjusted) analysis of the overall measure of household infrastructure, and four out of the five childhood illnesses included in this study, points to the importance of the general state of household infrastructure across a range of childhood illnesses.

Indicators of the socio-demographic environment, carer's psychosocial status and of health related behaviour showed associations with reported occurrence of a number of the childhood illnesses, although these associations were in general not as strong as the association with the measure of functional state of infrastructure. These findings point to the potential importance of interventions which target factors which impact negatively on the psychosocial status of carers and which target health related behaviour, including maintenance of household and personal hygiene. Interestingly, crowding was not independently associated with any of the child health outcomes, possibly due to almost universally high levels of crowding (90% of study houses had 3 or more *adults *compared to a national average total household size of 2.7 people) [48]. With regard to psychosocial factors, the indicator that showed the most consistent association in our analysis is factor 2 from the NLES. This factor related to the carer or household residents being concerned about someone involved in gambling, a serious accident, trouble with the police, being sent to jail, or being subject to racism. In contrast to factor 1 which relates to interpersonal violence and drug and alcohol abuse, factor 2 relates to issues of policing, injury, gambling and racism. In relation to health related behaviour and hygiene, there is increasing evidence for, and increasing program attention to, hand washing with soap in preventing disease [28,49-51]. These findings point to the potential importance of programs which enhance appropriate community policing, which discourage gambling and racism [52-56], and which target household hygiene. There is a need to strengthen the evidence base to support the development of effective interventions in these areas, at least in this study setting [56].

This study aimed to address one of the major recognised limitations of previous housing and health research - namely the measurement and assessment of the concurrent influence of a range of other related factors with the potential to confound or modify the association between housing condition and health. We also aimed to follow the principles of the MEME model by measuring a range of important exposures as well as a range of outcomes [31]. This was to overcome the limitations of much housing and health related research which focuses on specific exposures and specific outcomes [30]. Such studies are limited in terms of informing housing interventions which aim to achieve broad based health improvement through broad based housing improvements - either through new houses or extensive renovation.

A major strength of this study is the detailed assessment of the functional state of a wide range of items of housing infrastructure and of the hygienic condition of the household environment. Furthermore, the inclusion of multiple communities spread across a wide geographic area enhances the potential generalisability of the findings, at least within the context of remote Australian Aboriginal communities.

The study is subject to a number of limitations. First, the cross sectional design limits the potential to discern causative relationships, as the direction of influence between the factors is not clear. Second, some of the constructs represented in the conceptual framework are complex and may not be adequately represented by the indicators used in this study. Furthermore, potential important factors such as the quality of parenting [57] were not directly measured. Third, the measurement of a number of the indicators relies on face to face interviews with the carers of children and the main householder in each house. The large number of potential confounders of the association between house functional condition and child health increases the likelihood that some associations will be due to chance. This is to some extent unavoidable in the investigation of such a complex web of associations, but we have aimed to limit the potential for chance associations through the use of hierarchical models. Also relevant to this point is that a benefit of investigation of house function with a number of child health outcomes means that associations between primary explanatory variables and more than one of the outcomes of interest are less likely to be due to chance. Fourth, reporting on some of these indicators may be subject to respondent bias and/or misclassification. Fifth, outcome measurement relies on carers' report of childhood illness. While a two week recall period is considered to be less subjective to recall bias than longer recall periods, there is nevertheless potential for recall and respondent bias in the reporting of childhood illnesses. Reporting of childhood illness may also be subject to bias related to the relationship of the carer to the child, and the psychosocial state, education and language ability of the carer. The potential impact of these biases should be limited by the inclusion of relevant variables in the multivariate models. Considering that two week carer report for common childhood illness would be expected to show prevalence rates which are higher than for studies which rely on health service presentations or point in time clinical surveys, the high rates are consistent with other reports [1-10]. Finally, the odds ratios presented may over-estimate the strength of associations for high prevalence exposures.

## Conclusion

This study addresses an important gap in housing and health research, including in the specific context of remote Australian Aboriginal communities. The findings are relevant to current efforts to improve health through provision of improved housing [31] and confirm the potential for general improvements in the functional state of housing infrastructure to improve the health of children in these communities, most notably through reducing respiratory infections. The findings also support the evidence from a number of studies [32-34] which point to the need for building programs to be supported by a range of other social and behavioural interventions in order for the potential health gains of improved housing to be more fully realised.

## Abbreviations

BSD: Brief Screen for Depression; CDEP: Community Development Employment Program; CI: Confidence Interval; CRCAH: Co-operative Research Centre for Aboriginal Health; HICH study: Housing Improvement and Child Health study; HLPs: Healthy Living Practices; IHANT: Indigenous Housing Association of the Northern Territory; MEME: Multiple Exposure Multiple Effects; NHMRC: National Health and Medical Research Council; NLES: Negative Life Events Scale; OR: Odds Ratio.

## Competing interests

The authors declare that they have no competing interests.

## Authors' contributions

RB developed the study design and managed the implementation of the study. He developed the data analysis plan and played a primary role in the interpretation of study results and preparation of the manuscript. MS was responsible for data management and data analysis and contributed to preparation of the manuscript. EM developed and implemented the pilot study on which this study is based. She provided expert advice and support in contextualising study findings and assisted with manuscript preparation. DB and SG provided expert advice on data analysis and child health and participated in editing the manuscript. All authors read and approved the final manuscript.

## Pre-publication history

The pre-publication history for this paper can be accessed here:

http://www.biomedcentral.com/1471-2458/10/147/prepub

## Supplementary Material

Additional file 1 **Table 2a Socio-demographic variables unadjusted odds ratios (95% confidence interval) with carer report of child illness in previous two weeks. N = 618 children**. Socio-demographic variables and categories are listed and results provided according to illness categories: skin infection - no scabies; scabies w/wo infection; respiratory infection; diarrhoea and vomiting; ear infection. Click here for file

Additional file 2 **Table 2b Socio-economic status and financial stress variables and unadjusted odds ratios (95% confidence interval) for carer report of child illness in previous two weeks. N = 618 children**. Socio-economic and financial stress variables and categories are listed and results provided according to illness categories: skin infection - no scabies; scabies w/wo infection; respiratory infection; diarrhoea and vomiting; ear infection. Click here for file

Additional file 3 **Table 2c Psychosocial variables and unadjusted odds ratios (95% confidence interval) for carer report of child illness in previous two weeks. N = 618 children**. Psychosocial variables and categories are listed and results provided according to illness categories: skin infection - no scabies; scabies w/wo infection; respiratory infection; diarrhoea and vomiting; ear infection. Click here for file

Additional file 4 **Table 2d Health-related behaviour and hygienic state of environment variables and unadjusted odds ratios (95% confidence interval) for carer report of child illness in previous two weeks. N = 618 children**. Health-related behaviour and hygienic state of environment variables and categories are listed and results provided according to illness categories: skin infection - no scabies; scabies w/wo infection; respiratory infection; diarrhoea and vomiting; ear infection. Click here for file

Additional file 5 **Table 3 Primary explanatory variables unadjusted odds ratios (95% confidence interval) with carer report of child illness in previous two weeks. N = 618 children**. Primary explanatory variables (FHLP measure) are listed and results provided according to illness categories: skin infection - no scabies; scabies w/wo infection; respiratory infection; diarrhoea and vomiting; ear infection. Click here for file

Additional file 6 **Table 4 Multivariable adjusted models for carer report of child illness in previous two weeks**. Primary explanatory variables and categories (Specific HLP failed) and secondary explanatory variables (socio-demographic characteristics) are listed and results provided according to illness categories: skin infection - no scabies; scabies w/wo infection; respiratory infection; diarrhoea and vomiting; ear infection. Click here for file
