# Supplementary material for: Exploring cross-sectional associations between common childhood illness, housing and social conditions in remote Australian Aboriginal communities
Source: BMC Public Health. 2010 Mar 20;10:147. doi: 10.1186/1471-2458-10-147 (PMC2848201; doi:10.1186/1471-2458-10-147)
Supplement: Additional file 1 — Table 2a Socio-demographic variables unadjusted odds ratios (95% confidence interval) with carer report of child illness in previous two weeks. N = 618 children. Socio-demographic variables and categories are listed and results provided according to illness categories: skin infection - no scabies; scabies w/wo infection; respiratory infection; diarrhoea and vomiting; ear infection. [file 1471-2458-10-147-S1.DOC]

**Table 2a:** Socio-demographic variables unadjusted odds ratios (95% confidence interval) with carer report of child illness in previous two weeks. N=618 children

| **Socio-demographic variables** | **Variable categories** | **Missing**  **n (%)** | **Children**  **n (%)** | **Skin infection**  **no scabies**  **OR (95% CI)** | **Scabies w/wo**  **skin infection**  **OR (95% CI)** | **Respiratory**  **Infection**  **OR (95% CI)** | **Diarrhoea**  **& vomiting**  **OR (95% CI)** | **Ear**  **Infection**  **OR (95% CI)** |
| --- | --- | --- | --- | --- | --- | --- | --- | --- |
| Child age | <1 year  1-2 years  3-7 years | 0 (0.0) | 95 (15.4)  190 (30.7)  333 (53.9) | 1.00  **2.27 (1.05-4.90)**  **2.34 (1.09-5.03)** | 1.00  0.67 (0.37-1.22)  0.61 (0.34-1.10) | 1.00  1.00 (0.60-1.66)  0.64 (0.40-1.04) | 1.00  1.55 (0.93-2.60)  **0.50 (0.31-0.81)** | 1.00  **3.48 (1.91-6.32)**  1.77 (0.98-3.19) |
| Child sex | Male | 12 (1.9) | 289 (47.7) | 1.19 (0.78-1.80) | 1.03 (0.67-1.58) | **1.44 (1.01-2.07)** | **1.51 (1.07-2.14)** | 1.24 (0.87-1.77) |
| Child mobility | 10% or more in other dwelling | 0 (0.0) | 62 (10.0) | 1.50 (0.57-3.96) | 2.05 (0.77-5.46) | 1.59 (0.74-3.42) | 1.00 (0.57-1.76) | 0.68 (0.38-1.21) |
| Child attends day care | Attends day care (all children) | 5 (0.8) | 64 (10.4) | 1.17 (0.65-2.12) | 1.54 (0.81-2.93) | 1.04 (0.58-1.85) | 1.31 (0.73-2.33) | **2.35 (1.32-4.20)** |
| Child attends day care | Attends day care (children <6) | 5 (0.9) | 62 (11.7) | 1.17 (0.65-2.12) | 1.54 (0.81-2.93) | 1.04 (0.58-1.85) | 1.31 (0.73-2.33) | **2.35 (1.32-4.20)** |
| Carer age | Less than 20 years  20 to 34 years  35 plus years | 49 (7.9) | 38 (6.7)  379 (66.6)  152 (26.7) | 1.00  1.55 (0.68-3.53)  1.16 (0.65-2.07) | 1.00  1.68 (0.73-3.87)  1.50 (0.90-2.53) | 1.00  1.15 (0.55-2.42)  1.43 (0.91-2.23) | 1.00  2.01 (0.98-4.11)  1.18 (0.76-1.84) | 1.00  0.78 (0.38-1.59)  1.12 (0.71-1.77) |
| Carer lives with spouse | Carer lives with spouse  Carer does not live with spouse  Don’t know/refused | 1 (0.2) | 133 (21.6)  376 (61.0)  108 (17.5) | 1.00  1.21 (0.67-2.16)  0.97 (0.44-2.16) | 1.00  0.52 (0.30-0.90)  1.32 (0.67-2.59) | 1.00  0.95 (0.58-1.54)  1.01 (0.53-1.93) | 1.00  0.63 (0.39-1.02)  0.80 (0.42-1.50) | 1.00  0.97 (0.58-1.60)  1.19 (0.64-2.21) |
| Length of time in dwelling | Less than 1 year  1 to 3 years  3 to 5 years  5 or more years | 7 (1.1) | 109 (17.8)  122 (20.0)  104 (17.0)  276 (45.2) | 1.00  1.06 (0.51-2.23)  0.73 (0.34-1.56)  0.91 (0.49-1.69) | 1.00  1.50 (0.68-3.30)  1.06 (0.45-2.49)  1.25 (0.62-2.53) | 1.00  0.81 (0.45-1.47)  0.95 (0.51-1.76)  0.66 (0.38-1.14) | 1.00  0.56 (0.31-1.01)  0.73 (0.38-1.39)  0.88 (0.52-1.50) | 1.00  0.56 (0.30-1.07)  0.52 (0.27-1.01)  0.62 (0.36-1.06) |
| Crowding: Residents per bedroom quartiles^1^ | 1 to 2.33 persons per bedroom  2.34 to 3.25 persons per bedroom  3.26 to 4.00 persons per bedroom  4.01 to 11 persons per bedroom | 42 (6.8) | 139 (24.1)  155 (26.9)  130 (22.6)  152 (26.4) | 1.00  0.96 (0.51-1.81)  0.69 (0.33-1.44)  **1.95 (1.04-3.65)** | 1.00  1.11 (0.56-2.19)  1.04 (0.49-2.19)  0.78 (0.37-1.65) | 1.00  1.05 (0.58-1.91)  0.95 (0.51-1.79)  0.93 (0.55-1.58) | 1.00  1.50 (0.82-2.74)  1.19 (0.65-2.21)  1.53 (0.86-2.72) | 1.00  0.83 (0.47-1.47)  0.75 (0.41-1.37)  1.35 (0.79-2.32) |
| No. children$\leq$7 years living in dwelling | Less than 3 children in dwelling  Three or more children in dwelling | 6 (1.0) | 398 (65.0)  214 (35.0) | 1.00  0.86 (0.50-1.47) | 1.00  0.80 (0.48-1.33) | 1.00  0.84 (0.54-1.32) | 1.00  0.89 (0.59-1.34) | 1.00  0.71 (0.45-1.11) |
| Number of adults in dwelling | 1 or 2 adults  3 to 5 adults  6 to 7 adults  8 to 17 adults | 26 (4.2) | 64 (10.8)  219 (37.0)  171 (28.9)  138 (23.3) | 1.00  **2.92 (1.13-7.52)**  **2.76 (1.03-7.41)**  **3.96 (1.43-10.9)** | 1.00  1.00 (0.39-2.56)  1.70 (0.66-4.40)  2.08 (0.80-5.41) | 1.00  1.20 (0.62-2.32)  1.13 (0.55-2.30)  1.45 (0.74-2.85) | 1.00  1.03 (0.53-2.03)  1.57 (0.78-3.12)  1.25 (0.61-2.55) | 1.00  1.66 (0.77-3.60)  1.11 (0.51-2.45)  1.67 (0.73-3.81) |
| Child relationship to householder | Son/Daughter  Grandson/Grand-daughter  Niece/Nephew  Other | 0 (0.0) | 183 (29.6)  308 (49.8)  97 (15.7)  30 (4.9) | 1.00  **1.95 (1.09-3.48)**  1.83 (0.85-3.95)  2.02 (0.68-6.01) | 1.00  **2.31 (1.30-4.11)**  2.10 (0.97-4.54)  0.96 (0.25-3.63) | 1.00  **1.72 (1.09-2.72)**  1.73 (0.93-3.24)  1.85 (0.74-4.58) | 1.00  **1.98 (1.25-3.13)**  1.34 (0.73-2.44)  1.48 (0.63-3.47) | 1.00  1.13 (0.72-1.75)  1.45 (0.79-2.66)  0.43 (0.15-1.26) |
| Carer relationship to child | Mother  Father  Aunt  Grand-mother  Other | 9 (1.5) | 442 (72.6)  23 (3.8)  48 (7.9)  84 (13.8)  12 (2.0) | 1.00  0.59 (0.17-2.08)  0.78 (0.33-1.87)  1.14 (0.54-2.42)  0.78 (0.17-3.51) | 1.00  0.44 (0.10-1.93)  0.66 (0.19-2.24)  1.25 (0.69-2.29)  - | 1.00  0.36 (0.11-1.21)  0.55 (0.20-1.51)  1.33 (0.79-2.24)  0.80 (0.24-2.62) | 1.00  0.73 (0.28-1.91)  0.41 (0.17-1.01)  0.83 (0.49-1.40)  1.03 (0.25-4.22) | 1.00  0.91 (0.31-2.62)  0.76 (0.34-1.70)  1.28 (0.73-2.25)  0.85 (0.16-4.68) |

Note: All data presented is from Carer and/or Householder interviews unless otherwise indicated

^1^ Number of residents (includes visitors) reported by householder and carer. Number of bedrooms based on surveyor observation
