# Supplementary material for: Exploring cross-sectional associations between common childhood illness, housing and social conditions in remote Australian Aboriginal communities
Source: BMC Public Health. 2010 Mar 20;10:147. doi: 10.1186/1471-2458-10-147 (PMC2848201; doi:10.1186/1471-2458-10-147)
Supplement: Additional file 2 — Table 2b Socio-economic status and financial stress variables and unadjusted odds ratios (95% confidence interval) for carer report of child illness in previous two weeks. N = 618 children. Socio-economic and financial stress variables and categories are listed and results provided according to illness categories: skin infection - no scabies; scabies w/wo infection; respiratory infection; diarrhoea and vomiting; ear infection. [file 1471-2458-10-147-S2.DOC]

**Table 2b:** Socio-economic status and financial stress variables and unadjusted odds ratios (95% confidence interval) for carer report of child illness in previous two weeks. N=618 children

| **Socio-economic and financial stress variables** | **Variable categories** | **Missing**  **n (%)** | **Children**  **n (%)** | **Skin infection**  **no scabies**  **OR (95% CI)** | **Scabies w/wo**  **skin infection**  **OR (95% CI)** | **Respiratory**  **Infection**  **OR (95% CI)** | **Diarrhoea**  **& vomiting**  **OR (95% CI)** | **Ear**  **Infection**  **OR (95% CI)** |
| --- | --- | --- | --- | --- | --- | --- | --- | --- |
| Carer highest schooling | Grades 11 and 12  Grades 9 and 10  Grade 8 and below | 0 (0.0) | 225 (36.4)  319 (51.6)  74 (12.0) | 1.00  1.39 (0.86-2.25)  **2.30 (1.11-4.77)** | 1.00  1.38 (0.81-2.37)  0.78 (0.33-1.86) | 1.00  1.15 (0.74-1.79)  1.32 (0.71-2.45) | 1.00  1.10 (0.72-1.69)  1.09 (0.59-2.00) | 1.00  1.07 (0.69-1.64)  0.91 (0.48-1.72) |
| Carer labour force status | Employed in mainstream economy  Employed by CDEP^1^  Unemployed (First Start)  Pensioner (old age or sickness)  Parenting payment | 5 (0.8) | 41 (6.7)  104 (17.0)  266 (43.4)  17 (2.8)  185 (30.2) | 1.00  0.43 (0.16-1.14)  0.50 (0.21-1.21)  0.41 (0.07-2.62)  **0.37 (0.15-0.92)** | 1.00  1.54 (0.64-3.69)  0.71 (0.32-1.56)  2.02 (0.55-7.50)  1.13 (0.50-2.58) | 1.00  1.26 (0.59-2.66)  1.22 (0.64-2.30)  2.17 (0.77-6.11)  1.28 (0.64-2.56) | 1.00  1.33 (0.56-3.15)  1.00 (0.45-2.25)  0.74 (0.14-3.92)  1.10 (0.48-2.52) | 1.00  1.88 (0.77-4.62)  1.34 (0.59-3.06)  0.47 (0.05-4.42)  1.39 (0.61-3.18) |
| Material wealth^2^ | Neither phone/fridge working  Either phone/fridge working but not both  Phone and fridge working | 57 (9.2) | 162 (28.9)  309 (55.1)  90 (16.0) | 1.00  1.04 (0.58-1.86)  0.97 (0.43-2.17) | 1.00  0.92 (0.52-1.64)  1.02 (0.50-2.09) | 1.00  1.10 (0.68-1.79)  1.05 (0.58-1.89) | 1.00  0.92 (0.59-1.43)  **0.51 (0.27-0.96)** | 1.00  0.90 (0.55-1.46)  1.24 (0.66-2.34) |
| Householder community status^3^ | Less than four important positions  Four or more important positions | 15 (2.4) | 512 (84.9)  91 (15.1) | 1.00  1.11 (0.58-2.15) | 1.00  1.09 (0.61-1.94) | 1.00  0.92 (0.55-1.54) | 1.00  1.09 (0.66-1.79) | 1.00  1.17 (0.73-1.87) |
| Number of things done if ran out of money | Did not run out of money  Ran out of money (no action money/credit)  1-2 specific actions to get money / credit  3 or more actions to get money / credit | 11 (1.8) | 190 (31.3)  12 (2.0)  197 (32.5)  208 (34.3) | 1.00  2.01 (0.41-9.99)  1.69 (0.93-3.05)  1.67 (0.93-2.99) | 1.00  0.47 (0.05-4.20)  0.99 (0.56-1.76)  1.26 (0.71-2.24) | 1.00  0.30 (0.03-2.68)  1.25 (0.79-1.99)  **1.79 (1.11-2.90)** | 1.00  0.74 (0.20-2.76)  0.78 (0.48-1.26)  1.20 (0.76-1.90) | 1.00  1.40 (0.34-5.82)  1.17 (0.73-1.86)  1.08 (0.68-1.73) |
| Could raise $2000 in a week for emergency | Couldn’t raise $2000 in a week  Could raise $2000 in a week  Don’t know whether could raise $2000 | 4 (0.7) | 421 (68.6)  130 (21.2)  63 (10.3) | 1.00  0.76 (0.43-1.34)  0.79 (0.40-1.57) | 1.00  0.90 (0.51-1.60)  0.88 (0.42-1.88) | 1.00  1.07 (0.66-1.75)  1.77 (0.97-3.25) | 1.00  1.25 (0.80-1.96)  1.32 (0.68-2.54) | 1.00  1.06 (0.66-1.71)  0.87 (0.45-1.69) |

Note: All data presented is from Carer and/or Householder interviews unless otherwise indicated

^1^ CDEP: Community Development Employment Program, a type of ‘work for the dole’ program.

^2^ Functioning phone reported by householder, functioning fridge observed and tested by surveyor

^3^ Number of important positions held in the community by householder (e.g. member of housing committee, community council, regional or local health board etc.).
