# Supplementary material for: Exploring cross-sectional associations between common childhood illness, housing and social conditions in remote Australian Aboriginal communities
Source: BMC Public Health. 2010 Mar 20;10:147. doi: 10.1186/1471-2458-10-147 (PMC2848201; doi:10.1186/1471-2458-10-147)
Supplement: Additional file 3 — Table 2c Psychosocial variables and unadjusted odds ratios (95% confidence interval) for carer report of child illness in previous two weeks. N = 618 children. Psychosocial variables and categories are listed and results provided according to illness categories: skin infection - no scabies; scabies w/wo infection; respiratory infection; diarrhoea and vomiting; ear infection. [file 1471-2458-10-147-S3.DOC]

**Table 2c:** Psychosocial variables and unadjusted odds ratios (95% confidence interval) for carer report of child illness in previous two weeks. N=618 children

| **Psychosocial variables** | **Variable categories** | **Missing**  **n (%)** | **Children**  **n (%)** | **Skin infection**  **no scabies**  **OR (95% CI)** | **Scabies w/wo**  **skin infection**  **OR (95% CI)** | **Respiratory**  **Infection**  **OR (95% CI)** | **Diarrhoea**  **& vomiting**  **OR (95% CI)** | **Ear**  **Infection**  **OR (95% CI)** |
| --- | --- | --- | --- | --- | --- | --- | --- | --- |
| Carer could get help if in trouble^1^ | None  One or more | 4 (0.7) | 203 (33.1)  411 (66.9) | 1.00  1.23 (0.75-2.02) | 1.00  0.78 (0.48-1.26) | 1.00  1.18 (0.77-1.79) | 1.00  1.24 (0.83-1.85) | 1.00  1.38 (0.91-2.09) |
| Frequency of visits by carer to traditional lands | Lives-on  5+  2-4  LE1 | 13 (2.1) | 249 (41.2)  72 (11.9)  43 (7.1)  241 (39.8) | 1.00  0.83 (0.39-1.78)  0.51 (0.20-1.32)  0.65 (0.39-1.07) | 1.00  1.19 (0.55-2.57)  1.63 (0.73-3.63)  1.17 (0.69-1.99) | 1.00  0.85 (0.42-1.71)  1.70 (0.76-3.79)  0.84 (0.54-1.31) | 1.00  1.24 (0.68-2.27)  1.06 (0.46-2.43)  0.83 (0.54-1.27) | 1.00  0.73 (0.39-1.36)  1.06 (0.49-2.29)  0.76 (0.49-1.17) |
| Negative life events (factor 1) ^2^ | None  1 or more | 7 (1.1) | 149 (24.4)  462 (75.6) | 1.00  1.57 (0.91-2.73) | 0.69 (0.40-1.17) | 1.00  0.94 (0.60-1.48) | 1.00  1.47 (0.91-2.36) | 1.00  1.36 (0.82-2.24) |
| Negative life events (factor 2) ^2^ | None  1 or more | 10 (1.6) | 184 (30.3)  424 (69.7) | 1.00  1.71 (0.97-3.00) | 0.99 (0.59-1.67) | 1.00  **1.92 (1.24-2.97)** | **1.93 (1.21-3.07)** | 1.00  1.48 (0.96-2.27) |
| Negative life events (factor 3) ^2^ | None  1 or more | 6 (1.0) | 69 (11.3)  543 (88.7) | 1.00  2.35 (0.97-5.70) | 0.99 (0.48-2.07) | 1.00  1.27 (0.67-2.4.) | **2.57 (1.24-5.35)** | 1.00  1.34 (0.70-2.55) |
| Carer Brief Screen for Depression (BSD) | Not depressed  Depressed (BSD score 25+) | 20 (3.2) | 497 (83.1)  101 (16.9) | 1.00  1.21 (0.66-2.21) | 1.47 (0.81-2.68) | 1.00  **1.81 (1.13-2.91)** | 1.00  1.46 (0.91-2.34) | 1.00  1.38 (0.81-2.37) |

Note: All data presented is from Carer and/or Householder interviews unless otherwise indicated

^1^ Types of people: friend, neighbour, relative, work colleague, community agency, and professional

^2^ Number of specified items in this factor identified as a worry for the carer or another household resident (factor 1 includes alcohol problems, drug problems, witness to fighting, victim of threatened or physical violence, vandalism problems; factor 2 consisted of gambling problems, serious accident, family member in jail, trouble with police, and racism; and factor 3 consisted of death of a family member, sickness or chronic disability, and overcrowding)
