# Supplementary material for: Exploring cross-sectional associations between common childhood illness, housing and social conditions in remote Australian Aboriginal communities
Source: BMC Public Health. 2010 Mar 20;10:147. doi: 10.1186/1471-2458-10-147 (PMC2848201; doi:10.1186/1471-2458-10-147)
Supplement: Additional file 4 — Table 2d Health-related behaviour and hygienic state of environment variables and unadjusted odds ratios (95% confidence interval) for carer report of child illness in previous two weeks. N = 618 children. Health-related behaviour and hygienic state of environment variables and categories are listed and results provided according to illness categories: skin infection - no scabies; scabies w/wo infection; respiratory infection; diarrhoea and vomiting; ear infection. [file 1471-2458-10-147-S4.DOC]

**Table 2d:** Health-related behaviour and hygienic state of environment variables and unadjusted odds ratios (95% confidence interval) for carer report of child illness in previous two weeks. N=618 children

| **Health-related behaviour and hygiene variables** | **Variable categories** | **Missing**  **n (%)** | **Children**  **n (%)** | **Skin infection**  **no scabies**  **OR (95% CI)** | **Scabies w/wo**  **skin infection**  **OR (95% CI)** | **Respiratory**  **Infection**  **OR (95% CI)** | **Diarrhoea**  **& vomiting**  **OR (95% CI)** | **Ear**  **Infection**  **OR (95% CI)** |
| --- | --- | --- | --- | --- | --- | --- | --- | --- |
| Household cleaning equipment^1^ | All items present  At least one of the broom/mop/bucket absent | 76 (12.3) | 346 (63.8)  196 (36.2) | 1.00  1.03 (0.60-1.76) | 1.00  0.80 (0.47-1.36) | 1.00  1.03 (0.66-1.61) | 1.00  0.94 (0.62-1.43) | 1.00  1.06 (0.70-1.62) |
| No soap in dwelling^1^ | Soap present  No soap in bathroom, kitchen or laundry sinks | 46 (7.4) | 318 (55.6)  254 (44.4) | 1.00  1.14 (0.69-1.88) | 1.00  1.26 (0.77-2.07) | 1.00  1.46 (0.97-2.20) | 1.00  **1.63 (1.10-2.41)** | 1.00  1.13 (0.75-1.70) |
| Child breastfeeding history | child was not breastfed  child was breastfeed | 6 (1.0) | 65 (10.6)  547 (89.4) | 1.00  1.10 (0.55-2.18) | 1.00  0.67 (0.33-1.33) | 1.00  **0.33 (0.18-0.59)** | 1.00  1.07 (0.60-1.92) | 1.00  0.85 (0.47-1.54) |
| Number of inside cigarette smokers | None  1-2  3 or more | 0 (0.0) | 66 (10.7)  325 (52.6)  227 (36.7) | 1.00  2.45 (0.92-6.55)  **2.85 (1.04-7.78)** | 1.00  **2.96 (1.17-7.50)**  **4.20 (1.63-10.8)** | 1.00  1.37 (0.75-2.50)  1.80 (0.96-3.38) | 1.00  1.84 (0.98-3.47)  **2.45 (1.28-4.70)** | 1.00  1.21 (0.60-2.45)  1.52 (0.74-3.13) |
| **Hygiene measures^2^** |  |  |  |  |  |  |  |  |
| Wash people | Good (scores 1 & 2)  Intermediate (scores 3 & 4)  Worst (scores 5, 6 & 7) | 35 (5.7) | 72 (12.4)  356 (61.1)  155 (26.6) | 1.00  1.87 (0.92-3.80)  1.75 (0.75-4.06) | 1.00  0.93 (0.48-1.82)  1.25 (0.61-2.57) | 1.00  1.27 (0.72-2.26)  1.77 (0.95-3.30) | 1.00  1.43 (0.79-2.58)  1.59 (0.82-3.06) | 1.00  1.63 (0.87-3.07)  1.46 (0.71-3.01) |
| Wash clothes & bedding | Good (scores 1 & 2)  Intermediate (scores 3 & 4)  Worst (scores 5, 6 & 7) | 35 (5.7) | 91 (15.6)  342 (58.7)  150 (25.7) | 1.00  1.18 (0.55-2.54)  1.17 (0.52-2.66) | 1.00  0.77 (0.40-1.49)  0.65 (0.30-1.42) | 1.00  1.65 (0.98-2.79)  1.79 (0.97-3.32) | 1.00  1.58 (0.90-2.77)  0.90 (0.47-1.74) | 1.00  1.29 (0.77-2.18)  1.00 (0.52-1.93) |
| Food preparation & storage | Good (scores 1 & 2)  Intermediate (scores 3 & 4)  Worst (scores 5, 6 & 7) | 35 (5.7) | 96 (16.5)  303 (52.0)  184 (31.6) | 1.00  1.21 (0.67-2.18)  1.39 (0.73-2.66) | 1.00  0.69 (0.35-1.37)  0.99 (0.47-2.06) | 1.00  1.66 (0.95-2.88)  1.77 (0.99-3.17) | 1.00  **2.06 (1.13-3.75)**  1.77 (0.92-3.40) | 1.00  1.43 (0.88-2.34)  0.91 (0.52-1.61) |
| Remove human waste | Good (scores 1 & 2)  Intermediate (scores 3 & 4)  Worst (scores 5, 6 & 7) | 35 (5.7) | 81 (13.9)  355 (60.9)  147 (25.2) | 1.00  **1.97 (1.02-3.81)**  1.67 (0.80-3.49) | 1.00  0.55 (0.30-1.01)  0.63 (0.31-1.27) | 1.00  1.11 (0.66-1.88)  1.48 (0.83-2.65) | 1.00  1.24 (0.67-2.29)  1.11 (0.56-2.20) | 1.00  0.93 (0.55-1.58)  1.15 (0.63-2.10) |
| Remove rubbish | Good (scores 1 & 2)  Intermediate (scores 3 & 4)  Worst (scores 5, 6 & 7) | 34 (5.5) | 78 (13.4)  328 (56.2)  178 (30.5) | 1.00  1.78 (0.90-3.52)  1.78 (0.83-3.82) | 1.00  0.67 (0.33-1.35)  1.22 (0.59-2.52) | 1.00  1.36 (0.77-2.40)  1.69 (0.91-3.13) | 1.00  1.16 (0.66-2.06)  1.59 (0.85-2.98) | 1.00  1.64 (0.99-2.74)  1.21 (0.67-2.21) |
| Bedding & sleeping area | Good (scores 1 & 2)  Intermediate (scores 3 & 4)  Worst (scores 5, 6 & 7) | 35 (5.7) | 39 (6.7)  529 (90.7)  15 (2.6) | 1.00  3.11 (0.98-9.92)  3.00 (0.47-19.1) | 1.00  0.79 (0.31-2.01)  0.28 (0.03-2.90) | 1.00  2.26 (1.00-5.12)  1.38 (0.28-6.68) | 1.00  **2.47 (1.12-5.43)**  4.81 (0.85-27.3) | 1.00  2.22 (0.95-5.16)  **4.81 (1.14-20.3)** |
| Control temperature | Good (scores 1 & 2)  Intermediate (scores 3 & 4)  Worst (scores 5, 6 & 7) | 34 (5.5) | 47 (8.1)  427 (73.1)  110 (18.8) | 1.00  **4.43 (1.40-13.9)**  1.97 (0.55-7.05) | 1.00  0.53 (0.28-1.02)  0.73 (0.32-1.64) | 1.00  1.34 (0.68-2.66)  1.17 (0.52-2.63) | 1.00  1.48 (0.65-3.36)  1.40 (0.58-3.40) | 1.00  1.01 (0.49-2.09)  1.12 (0.48-2.60) |
| Control dust | Good (scores 1 & 2)  Intermediate (scores 3 & 4)  Worst (scores 5, 6 & 7) | 34 (5.5) | 67 (11.5)  380 (65.1)  137 (23.5) | 1.00  2.09 (0.97-4.50)  1.49 (0.65-3.42) | 1.00  0.70 (0.37-1.33)  1.27 (0.64-2.49) | 1.00  0.93 (0.52-1.67)  1.16 (0.60-2.23) | 1.00  1.62 (0.86-3.04)  1.48 (0.71-3.10) | 1.00  0.84 (0.45-1.56)  0.87 (0.43-1.78) |
| Household electricals | Good (scores 1 & 2)  Intermediate (scores 3 & 4)  Worst (scores 5, 6 & 7) | 34 (5.5) | 76 (13.0)  349 (59.8)  159 (27.2) | 1.00  0.83 (0.41-1.67)  1.10 (0.49-2.46) | 1.00  0.92 (0.43-1.95)  0.87 (0.37-2.00) | 1.00  1.64 (0.88-3.03)  1.35 (0.69-2.62) | 1.00  1.29 (0.66-2.54)  1.50 (0.74-3.06) | 1.00  1.10 (0.59-2.04)  0.91 (0.45-1.83) |
| Control mould | Good (scores 1 & 2)  Intermediate (scores 3 & 4)  Worst (scores 5, 6 & 7) | 35 (5.7) | 106 (18.2)  321 (55.1)  156 (26.8) | 1.00  1.21 (0.64-2.28)  1.05 (0.49-2.22) | 1.00  1.27 (0.64-2.52)  1.76 (0.87-3.58) | 1.00  0.91 (0.54-1.55)  1.13 (0.64-1.99) | 1.00  1.26 (0.75-2.13)  1.28 (0.71-2.29) | 1.00  0.80 (0.46-1.39)  0.81 (0.44-1.49) |
| House surrounds | Good (scores 1 & 2)  Intermediate (scores 3 & 4)  Worst (scores 5, 6 & 7) | 34 (5.5) | 61 (10.5)  283 (48.5)  240 (41.1) | 1.00  1.25 (0.55-2.85)  0.99 (0.42-2.33) | 1.00  1.04 (0.45-2.37)  1.44 (0.63-3.34) | 1.00  1.79 (0.89-3.62)  1.99 (0.97-4.06) | 1.00  1.81 (0.89-3.65)  1.55 (0.76-3.18) | 1.00  1.43 (0.70-2.93)  1.02 (0.48-2.17) |
| Separate animals & humans | Good (scores 1 & 2)  Intermediate (scores 3 & 4)  Worst (scores 5, 6 & 7) | 34 (5.5) | 67 (11.5)  384 (65.8)  133 (22.8) | 1.00  1.43 (0.71-2.90)  1.52 (0.66-3.51) | 1.00  **0.52 (0.28-1.00)**  0.48 (0.22-1.03) | 1.00  1.12 (0.63-1.99)  1.80 (0.93-3.47) | 1.00  1.21 (0.66-2.20)  1.21 (0.61-2.42) | 1.00  1.01 (0.54-1.90)  1.30 (0.64-2.62) |
| Control pests & vermin | Good (scores 1 & 2)  Intermediate (scores 3 & 4)  Worst (scores 5, 6 & 7) | 35 (5.7) | 69 (11.8)  282 (48.4)  232 (39.8) | 1.00  2.00 (0.87-4.59)  **2.82 (1.21-6.57)** | 1.00  0.57 (0.29-1.15)  1.05 (0.54-2.06) | 1.00  1.12 (0.52-2.43)  1.93 (0.89-4.17) | 1.00  1.19 (0.62-2.28)  1.64 (0.85-3.15) | 1.00  1.31 (0.68-2.52)  1.40 (0.72-2.73) |

Note: All data presented is from Carer and/or Householder interviews unless otherwise indicated

^1^ Observed by surveyor

^2^ Scored by surveyor on a 1 (best) to 7 (worst) likert scale
