# Supplementary material for: Exploring cross-sectional associations between common childhood illness, housing and social conditions in remote Australian Aboriginal communities
Source: BMC Public Health. 2010 Mar 20;10:147. doi: 10.1186/1471-2458-10-147 (PMC2848201; doi:10.1186/1471-2458-10-147)
Supplement: Additional file 5 — Table 3 Primary explanatory variables unadjusted odds ratios (95% confidence interval) with carer report of child illness in previous two weeks. N = 618 children. Primary explanatory variables (FHLP measure) are listed and results provided according to illness categories: skin infection - no scabies; scabies w/wo infection; respiratory infection; diarrhoea and vomiting; ear infection. [file 1471-2458-10-147-S5.DOC]

**Table 3**: Primary explanatory variables unadjusted odds ratios (95% confidence interval) with carer report of child illness in previous two weeks. N=618 children

| **Primary explanatory variables** | **Missing**  **n (%)** | **Children**  **n (%)** | **Skin infection**  **no scabies**  **OR (95% CI)** | **Scabies w/wo**  **skin infection**  **OR (95% CI)** | **Respiratory**  **Infection**  **OR (95% CI)** | **Diarrhoea**  **&/or vomiting**  **OR (95% CI)** | **Ear**  **Infection**  **OR (95% CI)** |
| --- | --- | --- | --- | --- | --- | --- | --- |
| *FHLP measure*^1^ |  |  |  |  |  |  |  |
| Wash people pass | 46 (7.4) | 94 (16.4) | 1.00 | 1.00 | 1.00 | 1.00 | 1.00 |
| Wash people fail |  | 478 (83.6) | 1.02 (0.51-2.04) | 1.68 (0.70-4.02) | 1.44 (0.83-2.50) | 1.37 (0.77-2.44) | 1.38 (0.82-2.32) |
| Wash clothes pass | 42 (6.8) | 167 (29.0) | 1.00 | 1.00 | 1.00 | 1.00 | 1.00 |
| Wash clothes fail |  | 409 (71.0) | 1.12 (0.62-2.01) | 1.32 (0.74-2.34) | 1.26 (0.77-2.05) | 0.96 (0.63-1.48) | 1.11 (0.73-1.70) |
| Prepare & store food pass | 39 (6.3) | 121 (20.9) | 1.00 | 1.00 | 1.00 | 1.00 | 1.00 |
| Prepare & store food fail |  | 458 (79.1) | 1.39 (0.77-2.49) | 0.96 (0.53-1.73) | 1.44 (0.91-2.27) | **1.70 (1.03-2.79)** | 1.25 (0.80-1.97) |
| Toilet pass | 46 (7.4) | 107 (18.7) | 1.00 | 1.00 | 1.00 | 1.00 | 1.00 |
| Toilet fail |  | 465 (81.3) | 1.32 (0.76-2.30) | 1.03 (0.61-1.73) | 1.19 (0.73-1.93) | 1.24 (0.76-2.03) | **2.26 (1.29-3.97)** |
| Remove waste water pass | 40 (6.5) | 396 (68.5) | 1.00 | 1.00 | 1.00 | 1.00 | 1.00 |
| Remove waste water fail |  | 182 (31.5) | 1.01 (0.61-1.65) | 1.33 (0.80-2.21) | 0.86 (0.55-1.34) | 0.83 (0.56-1.25) | 1.01 (0.65-1.58) |
| Remove rubbish pass | 35 (5.7) | 107 (18.4) | 1.00 | 1.00 | 1.00 | 1.00 | 1.00 |
| Remove rubbish fail |  | 476 (81.7) | 1.35 (0.66-2.80) | **0.54 (0.30-0.97)** | 1.06 (0.62-1.84) | 1.36 (0.77-2.42) | 0.91 (0.56-1.49) |
| Control dust (fence ) pass | 35 (5.7) | 166 (28.5) | 1.00 | 1.00 | 1.00 | 1.00 | 1.00 |
| Control dust (fence ) fail |  | 417 (71.5) | 1.36 (0.77-2.39) | **1.87 (1.03-3.39)** | 1.17 (0.74-1.84) | 1.25 (0.80-1.93) | 0.96 (0.62-1.48) |
| Reduce trauma pass | 34 (5.5) | 353 (60.5) | 1.00 | 1.00 | 1.00 | 1.00 | 1.00 |
| Reduce trauma fail |  | 231 (39.6) | 1.02 (0.63-1.67) | 1.38 (0.85-2.24) | 1.28 (0.85-1.93) | 1.14 (0.77-1.70) | 1.27 (0.84-1.91) |
| Number of HLPs failed |  |  |  |  |  |  |  |
| 0-2 | 49 (7.9) | 49 (8.6) | 1.00 | 1.00 | 1.00 | 1.00 | 1.00 |
| 3-8 |  | 520 (91.4) | 2.88 (0.78-10.6) | 1.27 (0.44-3.66) | 2.10 (0.96-4.56) | 2.42 (0.96-6.14) | **2.20 (1.08-4.46)** |

^1^ Infrastructure used to derive HLPs was observed and tested where required by the surveyor (see Table 1)
