# Supplementary material for: Exploring cross-sectional associations between common childhood illness, housing and social conditions in remote Australian Aboriginal communities
Source: BMC Public Health. 2010 Mar 20;10:147. doi: 10.1186/1471-2458-10-147 (PMC2848201; doi:10.1186/1471-2458-10-147)
Supplement: Additional file 6 — Table 4 Multivariable adjusted models for carer report of child illness in previous two weeks. Primary explanatory variables and categories (Specific HLP failed) and secondary explanatory variables (socio-demographic characteristics) are listed and results provided according to illness categories: skin infection - no scabies; scabies w/wo infection; respiratory infection; diarrhoea and vomiting; ear infection. [file 1471-2458-10-147-S6.DOC]

**Table 4:** Multivariable adjusted models for carer report of child illness in previous two weeks.

|  | **Model 1 (n=559)** | **Model 2 (n=617)** | **Model 3 (n=535)** | **Model 4 (n=551)** | **Model 5 (n=613)** |
| --- | --- | --- | --- | --- | --- |
|  | **Skin infection**  **no scabies**  **OR (95% CI)** | **Scabies w/wo**  **skin infection**  **OR (95% CI)** | **Respiratory**  **Infection**  **OR (95% CI)** | **Diarrhoea &/or**  **vomiting**  **OR (95% CI)** | **Ear**  **Infection**  **OR (95% CI)** |
| **Primary explanatory variables** |  |  |  |  |  |
| Specific Healthy Living Practices (HLP) failed | ns | ns | ns | ns | ns |
| Number of HLPs failed |  |  |  |  |  |
| 0-2 | ns | ns | 1.00 | ns | ns |
| 3-8 | ns | ns | **3.00 (1.36-6.63)** | ns | ns |
| **Secondary explanatory variables** |  |  |  |  |  |
| ***Socio-demographic*** |  |  |  |  |  |
| Childs age (years) |  |  |  |  |  |
| < 1 | ns | ns | **2.17 (1.25-3.76)** | **2.19 (1.28-3.75)** | 1.00 |
| 1-2 | ns | ns | 1.36 (0.87-2.14) | **3.09 (2.06-4.66)** | **3.18 (1.76-5.76)** |
| 3-7 | ns | ns | 1.00 | 1.00 | 1.59 (0.88-2.89) |
| Child gender |  |  |  |  |  |
| Male | ns | ns | ns | **1.59 (1.09-2.30)** | ns |
| Day care attendance |  |  |  |  |  |
| None | ns | ns | ns | ns | 1.00 |
| One or more days | ns | ns | ns | ns | **2.25 (1.26-3.99)** |
| Care lives with spouse |  |  |  |  |  |
| No, does not live with spouse | ns | 1.00 | ns | ns | ns |
| Yes, lives with spouse | ns | **0.53 (0.30-0.91)** | ns | ns | ns |
| Don’t know/refused | ns | 1.51 (0.77-2.97) | ns | ns | ns |
| ***Psychosocial*** |  |  |  |  |  |
| Carer NLES factor 2^§^ |  |  |  |  |  |
| One or more reported | ns | ns | ns | **1.78 (1.06-3.00)** | ns |
| Carer Brief Screen for Depression (BSD) |  |  |  |  |  |
| Depressed (25 or more) | ns | ns | **1.67 (1.01-2.77)** | ns | ns |
| ***Health-related behaviour and hygiene*** |  |  |  |  |  |
| Breastfeeding when child |  |  |  |  |  |
| Child was breastfeed | ns | ns | **0.27 (0.14-0.49)** | ns | ns |
| Presence of soap in house |  |  |  |  |  |
| Soap absent | ns | ns | ns | **1.67 (1.09-2.57)** | ns |
| *Hygiene measures* |  |  |  |  |  |
| Prepare and store food |  |  |  |  |  |
| Good (scores 1 & 2) | ns | ns | ns | 1.00 | ns |
| Intermediate (scores 3 & 4) | ns | ns | ns | **2.10 (1.10-4.00)** | ns |
| Worst (scores 5, 6 & 7) | ns | ns | ns | 1.51 (0.74-3.10) | ns |
| Control temperature |  |  |  |  |  |
| Good (scores 1 & 2) | 1.00 | ns | ns | ns | ns |
| Intermediate (scores 3 & 4) | **3.25 (1.06-9.94)** | ns | ns | ns | ns |
| Worst (scores 5, 6 & 7) | 1.41 (0.39-5.04) | ns | ns | ns | ns |
| Pests and vermin |  |  |  |  |  |
| Good (scores 1 & 2) | 1.00 | 2.01 (0.98-4.13) | ns | ns | ns |
| Intermediate (scores 3 & 4) | 1.96 (0.86-4.48) | 1.00 | ns | ns | ns |
| Worst (scores 5, 6 & 7) | **2.88 (1.25-6.60)** | **1.85 (1.08-3.16)** | ns | ns | ns |

Note: No significant interactions were observed in any of these models

ns Not significant (i.e. dropped out of model in backward selection process)

§ Negative Life Events Scale (NLES) factor 2 variable included: Gambling problems, serious accident, police troubles, family member sent to jail, racism
